# Unsupervised machine learning discovers classes in aluminium alloys

**DOI:** 10.1098/rsos.220360

**Published:** 2023-02-01

**Authors:** Ninad Bhat, Amanda S. Barnard, Nick Birbilis

**Affiliations:** College of Engineering and Computer Science, The Australian National University, Acton, ACT 2601, Australia

**Keywords:** alloy design, unsupervised learning, machine learning, aluminium, aluminium alloys, mechanical properties

## Abstract

Aluminium (Al) alloys are critical to many applications. Although Al alloys have been commercially widespread for over a century, their development has predominantly taken a trial-and-error approach. Furthermore, many discrete studies regarding Al alloys, often application specific, have precluded a broader consolidation of Al alloy classification. Iterative label spreading (ILS), an unsupervised machine learning approach, was used to identify the different classes of Al alloys, drawing from a specifically curated dataset of 1154 Al alloys (including alloy composition and processing conditions). Using ILS, eight classes of Al alloys were identified based on a comprehensive feature set under two descriptors. Further, a decision tree classifier was used to validate the separation of classes.

## 1. Introduction

Over the past decades, there has been an increase in interdisciplinary research involving machine learning (ML), in areas such as biology, agriculture, healthcare, climate and finance [1–7]. XGBoost regressors and convolutional neural networks have been used to predict the yield strength of additively manufactured metals with 90% accuracy [8]. Artificial neural networks have been used to predict glass-transition temperature with an *R*^2^ score of 0.998 [9]. Deep neural networks have been used to predict the likelihood of new crystal structures in Mn-Ge and Li-Mn-Ge systems [10].

Although databases regarding metallic alloys are accessible [11,12], the use of ML in alloy design has primarily been limited to supervised learning (SL) at this stage, and most have concentrated on developing structure–property relations [13–18]. SL uses a labelled feature set to design a model to predict the target output. This mapping of the features (structure) to the labels (properties) defines the relationship and enables predictions. The prediction of the material hardness of aluminium (Al) alloys [13] has previously been successfully carried out using gradient-boosted trees with an accuracy of 94%, obtained with a dataset of fewer than 1600 instances. Such structure–property relationships are often used to guide the design of new alloys; for example, designing new nickel-based alloys, as demonstrated by Conduit *et al*. [18].

Unsupervised learning (UL) uses only the unlabelled feature set to discover underlying patterns in the data, regardless of the properties. Compared with SL, there is an absence of studies employing UL in the context of metallic alloys design. Unsupervised word embedding was used in materials science to extract information from the literature [19], in a study that used information-dense word embedding to capture structure–property relations in materials. Clustering, a type of UL, partitions the dataset based on the similarity of instances. Clustering has been used to predict potential solid-state lithium conductors [20]. Such underlying patterns can be helpful in materials engineering to study relationships in alloy systems [21]. These clusters can further help rationalize large datasets and provide a basis for the physical understanding of subsequent SL models.

Understanding intrinsic patterns and relationships in Al alloys is of particular interest because of their utilization in the architecture, automotive and aerospace industries [22,23]. For example, the low-density and high-strength aluminium-lithium (Al-Li)-based alloys are used in the aircraft industry as a structural material [24]. Additionally, 5xxx-series (Al-Mg) and 6xxx-series (Al-Si-Mg) alloys are widely used in the automotive industry due to their high strength-to-weight ratio and corrosion resistance [25]. Laser additive manufacturing has also readily produced Al alloys [26,27]. Given the wide variety of uses of Al alloys and their ongoing potential for many applications owing to their high strength-to-weight ratio, designing new Al alloys becomes essential.

The design of new Al alloys can be performed by searching the feature space of possible alloy combinations and testing them. But due to the large feature space (number of processing conditions and alloying concentration), screening potential new Al alloys is difficult. Producing and testing each alloy in the feature space is cost intensive. The feature space can be reduced by identifying groups of similar properties and screening alloys within the group with the desired properties. Traditionally, Al alloys are separated into groups based on domain knowledge [28]. Using domain knowledge can introduce bias in ML. An alternative is to group Al alloys using clustering to group instances based on feature similarities. Screening candidates in a cluster is a more viable and financially sustainable materials design approach, as it naturally allows for a fault tolerance.

The present study involves a dataset including Al alloys with various processing conditions and concentrations of alloying elements. The dataset includes 1154 unique instances and is the largest known single dataset of its type (for Al alloys). This dataset was used for unsupervised ML to identify the different clusters of Al alloys based on concentration and processing conditions. The features leading to the alloy's categorization into different classes were also analysed using an interpretable supervised classifier.

## 2. Dataset and methods

### 2.1. Dataset

The dataset used in this study includes 1154 Al alloys from various literature sources (notably [29–31]) and unpublished data from the authors' laboratory [30]. Curation was not conducted as part of the present work, and the dataset may be accessed at [32]. The dataset contains unique entries for each alloy that comprises alloy composition, and the ‘Processing Type’ feature denotes the processing condition, providing a chemical descriptor and an engineering descriptor, respectively. The combination of the chemical descriptor and the encoded engineering descriptor provides the holistic alloy features for each alloy in the dataset. Table 1 shows that many processing conditions exist in Al-alloy production, the descriptions of which are well documented by the US Aluminum Association Alloy and Temper System [33].

**Table 1.** List of processing conditions and encoded Processing Type.

processing condition	Processing Type
AQ (As quenched), As fabricated, O (annealed), As cast	no processing
SHT (solutionized, or solution heat treated)	solutionized
H11, H111, H112, H116,H12, H131	strain hardened
H, H14, H16, H18, H19, H191, H24, H25, H26, H27, H28, H311, H32, H321, H323, H34, H343, H36, H38	strain hardened (hard)
T1, T14	naturally aged
T3, T31, T32, T33, T351, T3510, T511, T36, T361, T37, T39	solutionized + cold worked + naturally aged
T4, T41, T42, T451, T4510, T4511	solutionized + naturally aged
T5, T53	artificial aged
T6, T61, T6151, T61511, T62, T64, T651, T6510, T6511, T652, T66	solutionized + artificially peak aged
T7, T72, T73, T7351, T73510, T73511, T736, T73651, T74, T7451, T74511, T7452, T75, T76, T761, T7651, T76510, T76511, T773, T7751, T7751, T77511, T8, T81, T82, T83, T831, T832, T385, T84, T85, T851, T8510, T8511, T852, T86, T861, T87, T9, T91, T913	solutionized + artificially over aged

The composition spread (provided as alloying element concentration in wt. %) is given as a range for the elements present in the feature dataset used herein, as per table 2.

**Table 2.** Concentration range for elements in Al alloys. The concentration ranges are normalized and standardized during the pre-processing stage.

element	min. (wt. %)	max. (wt. %)
Ag	0	0.69
Al	0.7495	99.99
B	0	0.01
Be	0	0.0045
Bi	0	0.548
Cd	0	0.2
Co	0	0.8
Cr	0	4.11
Cu	0	6.85
Er	0	0.41
Eu	0	0.095
Fe	0	1.2
Ga	0	0.03
Li	0	3.82
Mg	0	6
Mn	0	1.31
Ni	0	2
Pb	0	0.054
Sc	0	1.45
Si	0	0.22
Sn	0	20
Ti	0	0.25
V	0	0.003
Zn	0	12
Zr	0	1.5

### 2.2. Methods

#### 2.2.1. Pre-processing

One-hot encoding represents categorical data using vectors containing binary values [34,35]. Each vector index denotes the existence of a category with ‘1’ and an absence of the category with ‘0’. One-hot encoding was used to represent the processing type feature as there is no ordinal relationship between the processes. One-hot encoding was carried out using OneHotEncoder in the sklearn package [36]. For example, processing type ‘Naturally aged’ is replaced by vector [0, 0, 0, 0, 1, 0, 0, 0, 0, 0]. After one-hot encoding, the processing type was represented in 10 feature columns.

Feature scaling prevents bias when descriptors are measured on different scales. The chemical (alloying concentrations) and engineering (processing type) descriptors have different scales, leading to bias in the ML model. Normalization and standardization of the dataset were carried out using MinMaxScaler in the sklearn package [36,37].

When training ML models, highly correlated features represent the same information, which might lead to bias. The pairwise correlation of features was calculated using Pearson's correlation coefficient [38]. Pb and Bi showed the highest correlation, with the value of Pearson's coefficient being 1. Hence, Pb was removed, while training the model as Bi has been shown to have a greater impact on the mechanical property of Al alloys [39] and due to environmental concerns about the use of Pb [40]. The next highest correlation of 0.81 was not high enough to be considered highly correlated. Correlations between other features can be found in the supporting information.

#### 2.2.2. Clustering

Clustering algorithms, a subset of unsupervised ML algorithms, identify subgroups in the dataset based on the similarity index of features with no prior assumptions [41,42]. In this study, iterative label spreading (ILS) has been used because it requires no hyperparameter optimization and can predict the number and type of clusters in the dataset before clustering (something that is required in advance for other label-spreading approaches) [43]. ILS has successfully been used in materials science applications [21,44–46]. ILS works better than other methods in clustering high-dimensional data with significant noise, is able to identify both concave and convex clusters, and can identify challenging cases such as the null case and the chain case where other methods fail [43].

For ILS clustering, the dataset is divided into a set of labelled points *L* (where *L* ≠ 0) and a set of unlabelled points *U*. The similarity index of two instances is calculated using a function, which may take the form of a Euclidean distance given by

2.1 $$D(x,\;y)=\;{\left(\overset{d}{\underset{i=0}{\sum }}{\left(x_{i}-y_{i}\right)}^{2}\right)}^{0.5},$$

where *x* and *y* both belong to *d*-dimensional space. As *L* is a non-empty set, one instance in the dataset is labelled manually before clustering. After labelling a point, ILS is run on the dataset. In each iteration, pairwise similarity indexes (Euclidean distances, in this case) are calculated between labelled points and unlabelled points. After this, the highest similarity index between labelled and unlabelled points is found. Let (*x′*, *y′*) denote the instances with the highest similarity (minimum distance *R*_min_), where *y′* ∈ *L* and *y′* ∈ *U*. Instance *y′* is labelled with *y′* and moved from set *U* to set *L*. In each iteration, one instance from the *U* is labelled and moved to *L*. The minimum distance (*R*_min_), *y′* and *x′* information are recorded for each iteration. The iterations continue till *U* is empty and all points are labelled.

The time taken to calculate the minimum pairwise distance increases quadratically with the increase in the dataset size. As ILS calculates *R*_min_ every iteration, the time taken to run ILS increases cubically with the dataset's size. This time complexity is higher than traditional methods like k-means and DBSCAN [41]. But the high time complexity of ILS did not factor into model selection due to the small dataset size.

To determine the number of clusters in the dataset, ILS is initialized with one labelled point. ILS labels each point iteratively and returns the minimum distances and the order in which the points were labelled. The ordered minimum distance plot is used to determine the number of clusters. Each peak in the plot denotes a density discontinuity. These peaks divide the plot into regions, each corresponding to a unique cluster. The number of peaks is counted using a continuous wavelet transform peak finding algorithm with smoothing over 30 points, implemented in SciPy Library [47]. On identifying distinct regions, one point from each region is relabelled with a unique label. ILS is rerun to determine the label of each instance in the dataset. ILS can also be applied individually to each cluster to find sub-clusters, confidently identifying all clusters without the need for hyperparameter optimization.

#### 2.2.3. Classification

Classification methods are supervised ML algorithms with discrete target variables in addition to features. Classifiers can determine if clusters represent separable classes. This study uses the nonlinear, non-parametric and interpretable decision tree classifier (DTC) [48]. A DTC predicts discrete class labels following decision rules based on Boolean logic and inferred from the features. DTCs are trained by recursively splitting the data and are able to handle multi-output problems. DTCs require little data preparation, can be validated using statistical tests, are interpretable and can be visualized. DTCs have the advantage of exposing the feature importance profile, allowing for model interpretability. The importance of each feature is equal to the total reduction in gini impurity due to the feature. The hyperparameters for DTC, optimized using grid search, are criterion = ‘gini’, splitter = ‘best’, max_depth = 8, min_samples_leaf = 1, max_features = None, ccp_alpha = 0, class_weight = None and random_state = 0.

#### 2.2.4. Projections

The t-distributed stochastic neighbour embedding (t-SNE) [49] was used for visualization of data in two dimensions. Specifically, t-SNE with a feature space of 34 dimensions and perplexity of 100 was used to project the dataset in two dimensions. Then, the resultant scatter plot was encoded by the cluster label.

#### 2.2.5. Calculation of phase concentrations

Secondary phase concentrations of Al alloys were calculated using CALPHAD (CALculation of PHAse Diagrams) in PANDAT [50,51]. PANDAT uses equilibrium thermodynamics to predict alloy phase fractions (for a given composition). The phase concentrations were calculated at 25° Celsius at equilibrium conditions.

## 3. Results

### 3.1. Clustering

**Figure 1.**
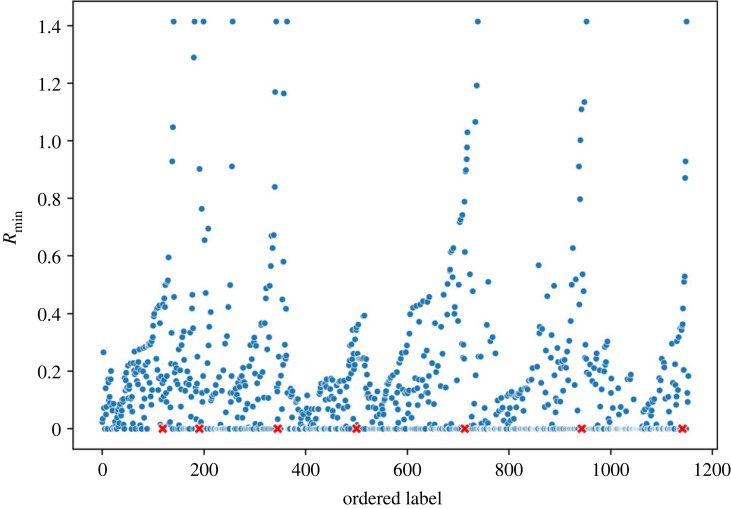
Ordered label versus *R*_min_ plot for Al-alloy dataset using ILS clustering. The red crosses denote the peak found using continuous wavelet transform peak finding algorithm.

**Figure 2.**
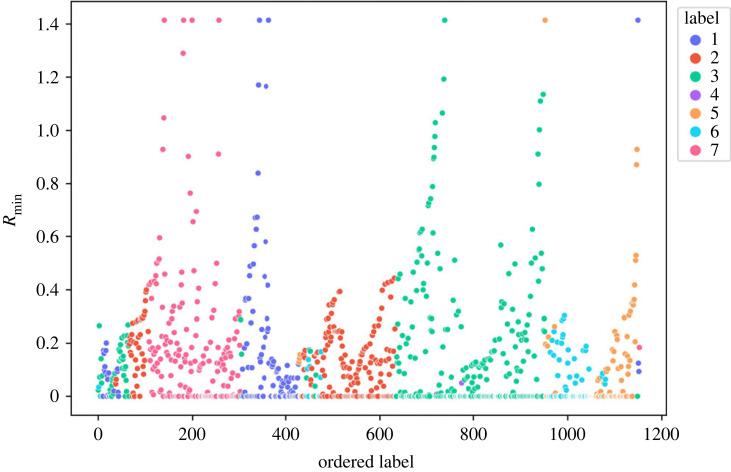
Minimum distance *R*_min_ versus ordered label after the second pass of ILS clustering for 1154 Al alloys. Each colour denotes a cluster associated with the initially labelled points.

The first pass of ILS was carried out after labelling a single point in the dataset. The resultant plot is shown in figure 1. Using a continuous wavelet transform peak finding algorithm with smoothing over 30 points, seven peaks, depicted by the red crosses in figure 1, were identified. These data instances were used as the labelled points for the second pass of ILS, as described in §2.2. The seven distinct clusters shown in figure 2 were then definitely identified, but Cluster 4 was removed as it was statistically insignificant with only three alloys in it. These three alloys can be considered as a group of similar outliers. The outliers detected were the only T6 alloy in the dataset that contained Bi as an alloying element.

**Figure 3.**
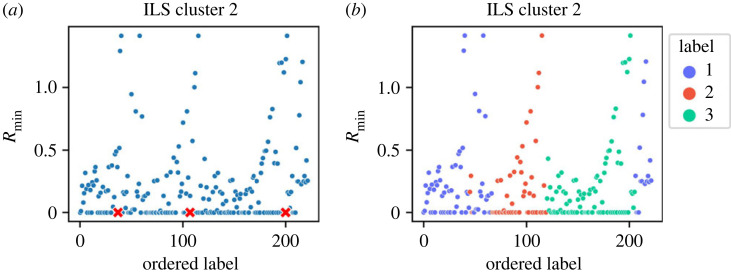
Order-labelled *R*_min_ plot for Cluster 2. (*a*) The first pass identifying three peaks proving the existence of sub-clusters. The red crosses denote the location of the three peaks identified. (*b*) Second pass of ILS clustering identifying the three sub-clusters.

**Figure 4.**
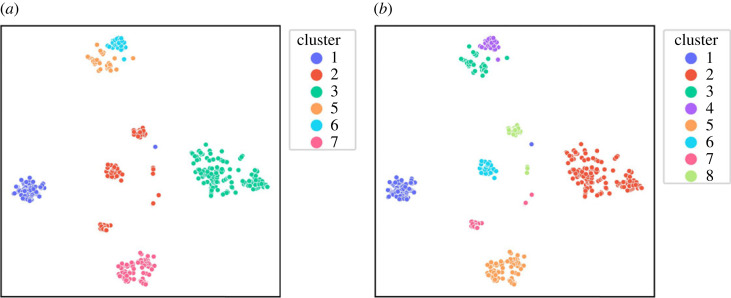
t-SNE map of 34-dimensional Al-alloy dataset encoded with ILS clustering labels. (*a*) t-SNE plot showing initial clustering results, (*b*) t-SNE plot with final labels after running ILS of each cluster.

Figure 2 suggests that sub-clusters might exist within the identified clusters hidden by the noise. On applying ILS to each cluster individually, we further identified three more clusters within Cluster 2 (shown in figure 3). Figure 4 shows the separation of Cluster 2 (in figure 4*a*) into Clusters 6, 7 and 8 (in figure 4*b*) using t-SNE plots. The application of ILS for each cluster is represented in the supporting information. After identifying all sub-clusters, the final clusters were labelled from 1 to 8. Cluster 1 can be seen in two regions in figure 4*b*. One of the regions covers ‘SHT’ alloys, while the others cover ‘As Cast’ alloys. Similarly, the two regions of Cluster 7 seen in figure 4*b* cover ‘Strain hardened’ and ‘Artificially aged’ alloys, respectively. The clustering results of ILS are compared with clustering results of k-means in the supplementary information.

### 3.2. Classification

**Figure 5.**
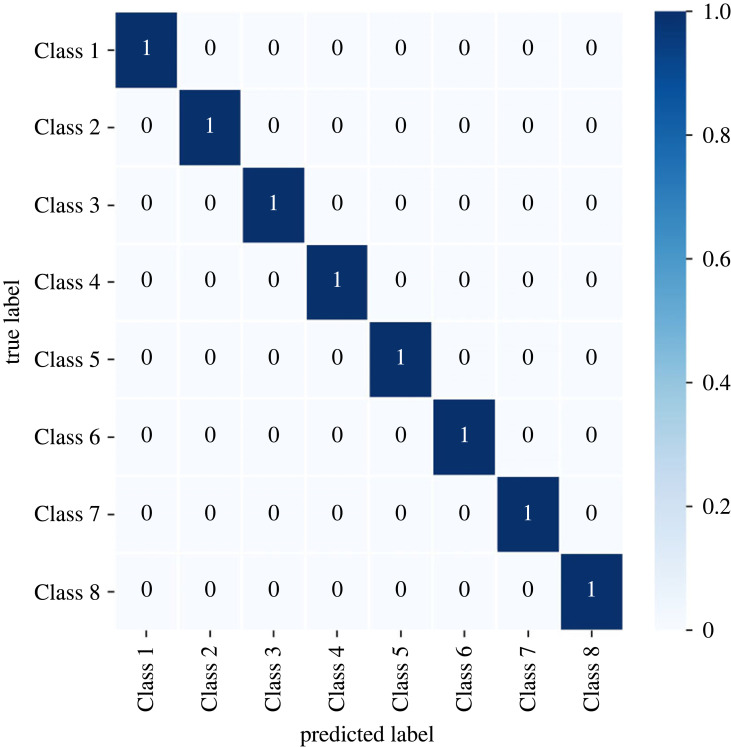
Confusion matrix showing true positive, true negative, false positive and false negative of DTC classes.

**Figure 6.**
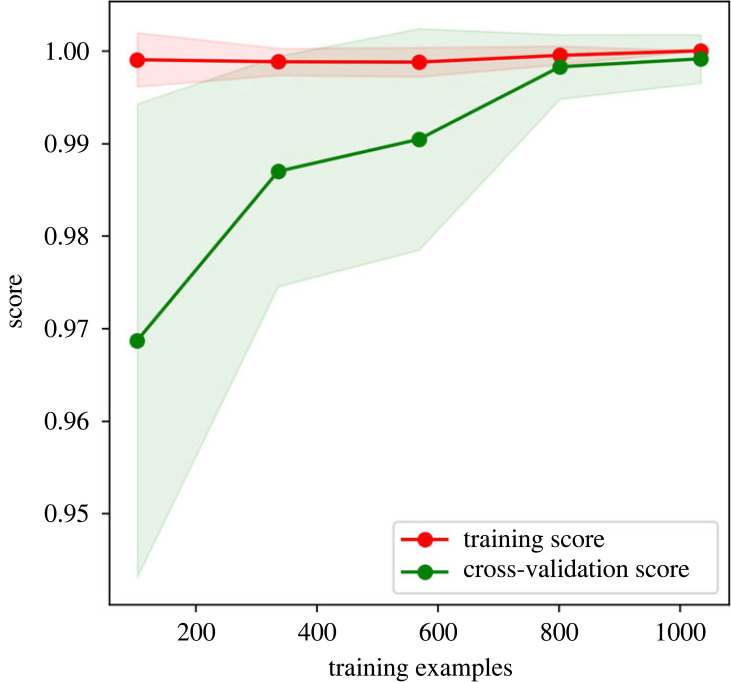
Learning curve for DTC showing high accuracy with limited training instances.

**Figure 7.**
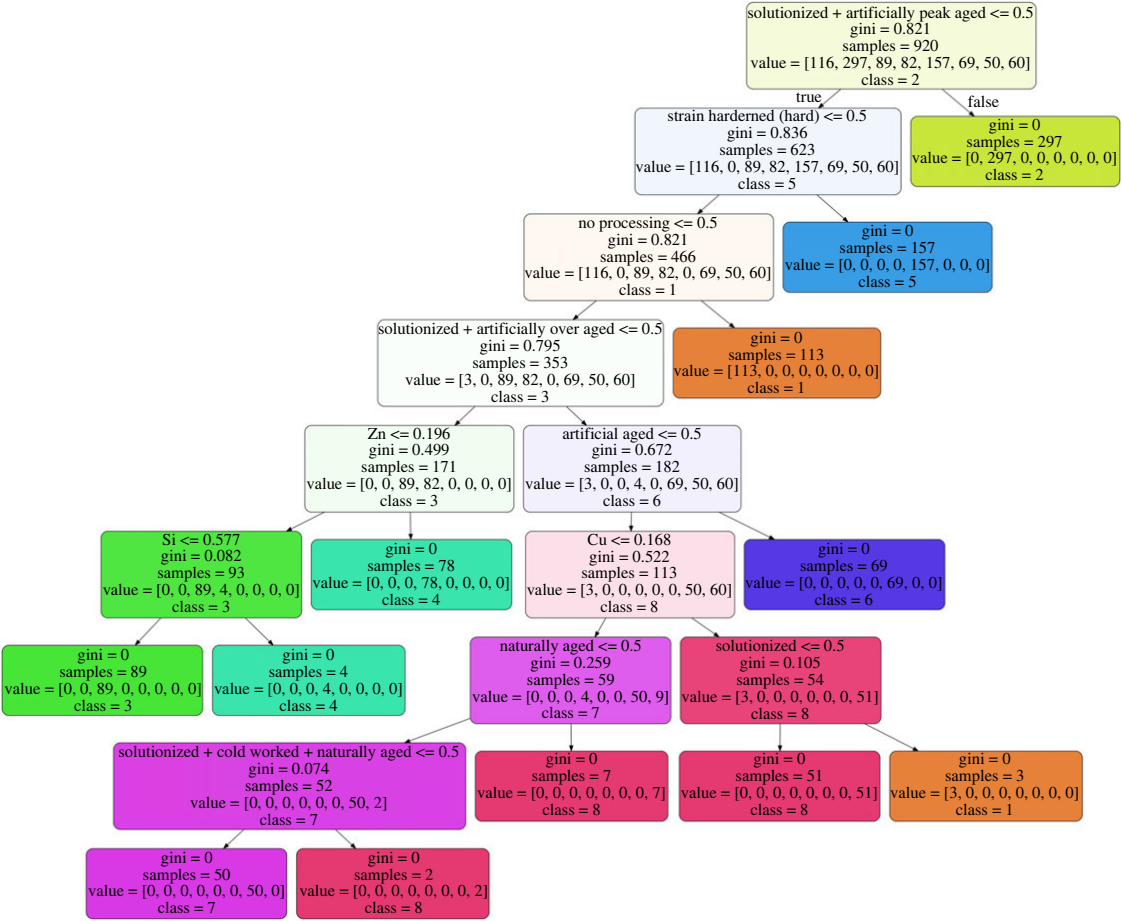
Decision tree trained on all features showing separation of eight classes. The decision tree shows gini impurity and dominant class at each node.

The DTC was used to determine if the clusters are separable classes, using the cluster number as labels. The classifier obtained a test *R*^2^ score of 1 and a fivefold cross-validation score of 0.994 ± 0.009. Table 3 shows the high precision, recall and f1-score of the DTC. The confusion matrix in figure 5 confirms that the clusters are classes. The learning curve in figure 6 demonstrates that the model exhibits low underfitting and overfitting for more than 800 training examples. The decision tree diagram is shown in figure 7; it shows the gini impurity and dominant class for each node and leaf. Each node in the decision tree shows the splitting criteria for the node. In the case of processing type, the splitting criteria verify the existence or absence of a processing type. The decision tree shows that the alloying concentration, apart from Zn, Cu and Si, do not play a part in determining the class of an alloy. This decision tree can be used to further classify new Al alloys into the eight classes.

**Table 3.** Classification report of DTC, showing the precision, recall and f1-score.

	precision	recall	F1-score
Class 1	1.0	1.0	1.0
Class 2	1.0	1.0	1.0
Class 3	1.0	1.0	1.0
Class 4	1.0	1.0	1.0
Class 5	1.0	1.0	1.0
Class 6	1.0	1.0	1.0
Class 7	1.0	1.0	1.0
Class 8	1.0	1.0	1.0

**Figure 8.**
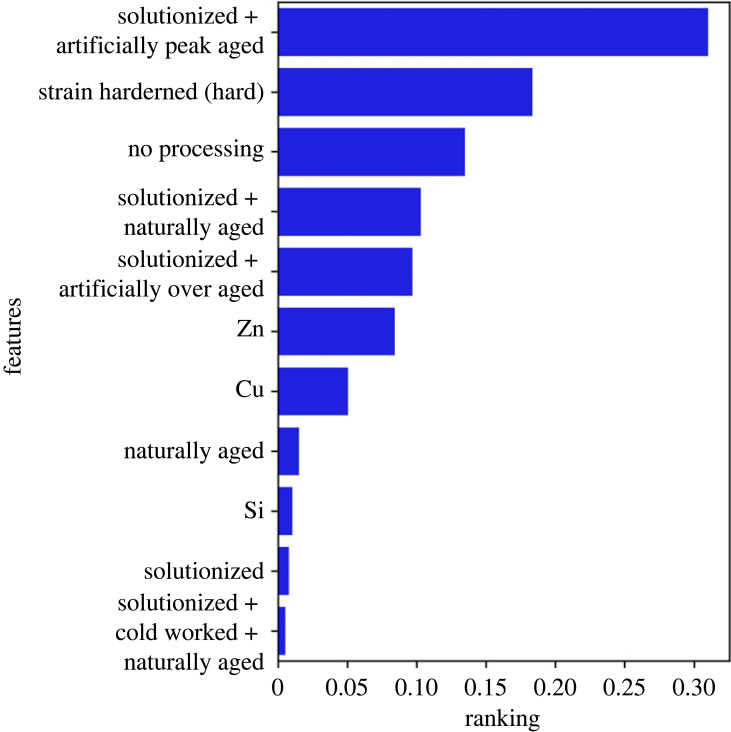
FIP of DTC showing the high importance of processing conditions in determining the class of Al alloys. FIP also shows that a small subset of features entirely determines classes.

**Figure 9.**
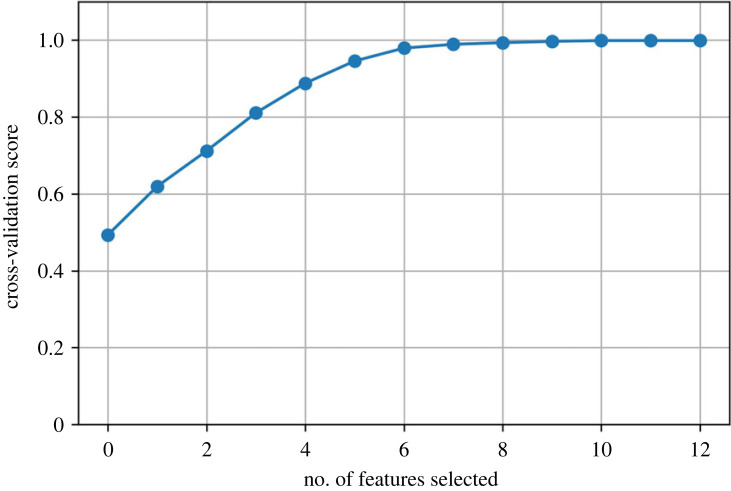
Recursive feature elimination to find the ideal number of features. A high cross-validation score can be achieved by only using 11 features.

**Figure 10.**
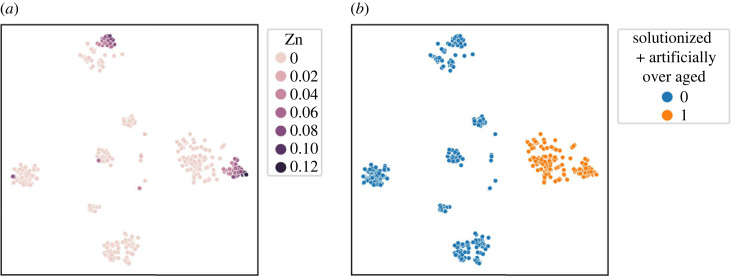
t-SNE plot encoded with features. (*a*) Encoded with highest importance concentration feature (Zn), (*b*) encoded with highest importance processing condition (solutionized + artificially over aged).

As mentioned above, an advantage of the DTC is that it also returns the relative importance of various features. Figure 8 shows the feature importance profile, which provides insights into how the model arrives at the prediction. These insights are not available from clustering algorithms directly. The results show that processing conditions play a significant role in deciding the class of an alloy, and the classes can be separated without using all the features. Recursive feature elimination, shown in figure 9, found that the ideal number of features is 10, which is considerably lower than using all the 34 features. The 11 features, listed in table 4, include eight processing types and alloy concentrations of Cu, Zn and Ti. A notable difference between the 11 features and the feature importance profile was the inclusion of Ti in place of Si. On retraining the models with these 11 features, we found no loss in the performance. The performance of the model trained on 11 features along with the decision tree is shown in the supplementary information. The dominant features were used to encode the t-SNE plot in figure 10. The most important processing condition and concentration feature are used for encoding, but similar plots can be calculated for other features.

**Table 4.** Eleven features calculated using recursive feature elimination.

feature	feature type
solutionized	Processing type
naturally aged	Processing type
solutionized + artificially over aged	Processing type
solutionized + naturally aged	Processing type
no processing	Processing type
strain hardened (hard)	Processing type
solutionized + artificially peak age	Processing type
solutionized + cold worked + naturally aged	Processing type
Cu	concentration
Zn	concentration
Ti	concentration

## 4. Discussion

As demonstrated by the results herein, UL indicates that the processing conditions play a vital role in determining the class of an alloy. Class 1 includes as-cast alloys. Class 2, Class 3 and Class 4 include solution-treated alloys that are artificially aged and overaged. Class 5 includes strain-hardened Al alloys. Class 6 contains alloys that are solution treated and then naturally aged. Class 7 includes artificially aged alloys. Finally, Class 8 includes naturally aged Al alloys.

Table 5 lists typical features of alloys in the DTC Classes and examples of the corresponding input alloys. A general inspection of the classes provides some readily apparent insights. For example:

— Class 8 includes the alloys with the highest Fe concentrations.
— Class 7 contains alloys with high Mg concentrations.
— Class 1 includes alloys that are ‘as cast’ or ‘solution heat treated’, regardless of the elemental concentrations.
— Class 2 includes alloys with high Cu concentrations.
— Class 5 includes alloys with low Cu concentrations.

**Table 5.** Class properties and alloy examples. Text in italics shows the traditional Al-alloy designation of examples.

Class	typical class properties (not exhaustive)	(some) example alloys
1	As cast, solution heat treated	Al-7.8Cu-0.19Mn-0.76Zn – SHT
	(typically) high Mn	Al-1Mg-0.6Si-0.28Cu-0.2Cr (*AA6061*) – O
		Al-4.4Mg-0.7Mn-0.15Cr (*AA5083*) – O
2	T6xx tempering,	Al-0.028Eu-2.8Mg-7.2Si – T6
	appreciable Cu	Al-1.2Cu-03.3Mg-12Zn – T6
	presence of Bi	Al-0.2Cr-0.27Cu-0.6Si (*AA6061*) – T6
		Al-0.22Cu-0.35Fe-1.1Mg-0.12Mn-0.58Si (*AA6061*) – T6
3	T9xx and most T8xx tempering	Al-0.2Cr-0.25Cu-0.6Si – T91
	some T7xx tempered alloys	Al-0.4 Ag-5.4Cu-1.3Li-0.14Zr – T8
	presence of Ag	
	presence of Ni	
4	T7xx	Al-2.3Cu-2.2Mg-6.2Zn (*AA7050*) – T73510
	some T8xx tempered alloys	Al-0.1Co-2.85Li – T7
5	Hxx tempering	Al-6Mg-0.6Mn – H14
	low Cu	Al-0.5Mg-0.6Mn – H14
	low Si	Al-0.1Cr-4.45Mg-0.8Mn – H321
	typically, appreciable Mg	
6	T4xx tempering	Al-0.2Cr-0.28Cu-0.35Fe-1Mg-0.075Mn-0.600Si-0.075Ti-0.125Zn (*AA6061*) – T4
	high Cr	
7	T5xx and Hxx	Al-0.7Mg-0.4Si – T5
	artificial aged	Al-0.12Er-1.2Mn – H12
	presence of Mg	
8	T3xx tempering	Al-0.1Cr-0.1Cu-0.35Fe-0.675Mg-0.4Si – T33
	high Fe	Al-1.3Cu-0.2Fe-2.5Li-0.7Mg – T351
	T1xx tempering	

The traditional Al-alloy designation system first classifies Al alloys based on manufacturing conditions; for example, wrought alloys and cast alloys. Each of the classifications is sub-classified based on major alloying elements. Wrought alloys have 8 Xxxx alloy series (sub-classes) based on the concentration of principal alloying elements. Similarly, cast alloys also have nine sub-classes. Each of these sub-classes is further divided depending on the heat treatment of the alloys. As such a classification is created by materials scientists, it is a multi-step process and is prone to observation and exclusion biases. The classification created through ILS uses both processing conditions and concentration simultaneously. Using both descriptors for classification leads to eight classes, which is considerably lower than 18+ sub-classes in the traditional designation. This classification is a single-step process and is data-driven, which reduces the chances of observation and exclusion biases.

**Figure 11.**
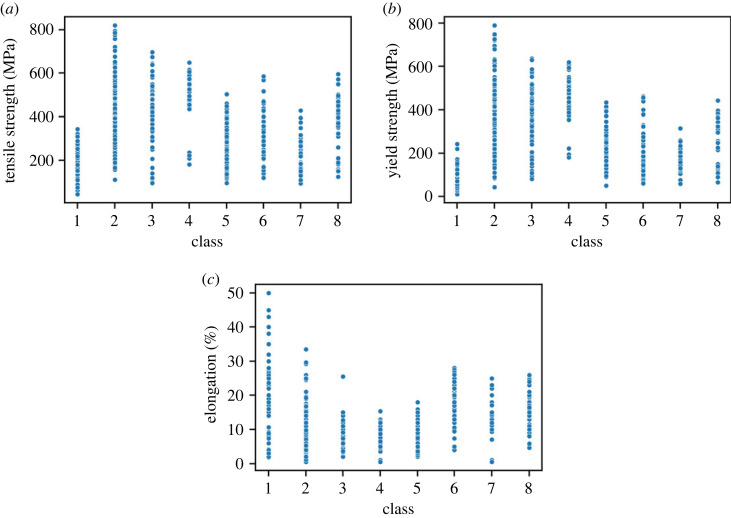
Range of mechanical properties for each ILS class. (*a*) Tensile strength (MPa), (*b*) yield strength (MPa) and (*c*) elongation (%).

The clustering and classification process pipeline presented in this paper (and summarized in the figures and tables above) is relevant to the rationalization of Al-alloy classes to date and can help in designing new Al alloys. This is emphasized by the data presented in figure 11, which maps the mechanical properties (figure 11*a* tensile strength, figure 11*b* yield strength and figure 11*c* elongation) of each of the alloys explored in this study to each of the eight DTC classes. Some classes cover a wider range of properties, while others ensure that mechanical properties lie in a smaller distribution. During alloy design, the variation of the properties in the class allows tuning of input parameters (concentration and processing condition) to achieve the desired alloy. What can be readily observed from figure 11*a* is that the alloys with the highest tensile strength (i.e. greater than 500 MPa) belong to Classes 2, 3 and 4. The highest strength alloy corresponds to Class 2. Similarly, relationships exist between classes in figure 11*c* for the Al-alloy elongation. Typically, strength and elongation are inversely proportional; however, what is evident in figure 11 is that there is a range of values within each class. The same is true for yield strength (figure 11*b*) with trends that mimic the tensile strength (figure 11*a*).

**Figure 12.**
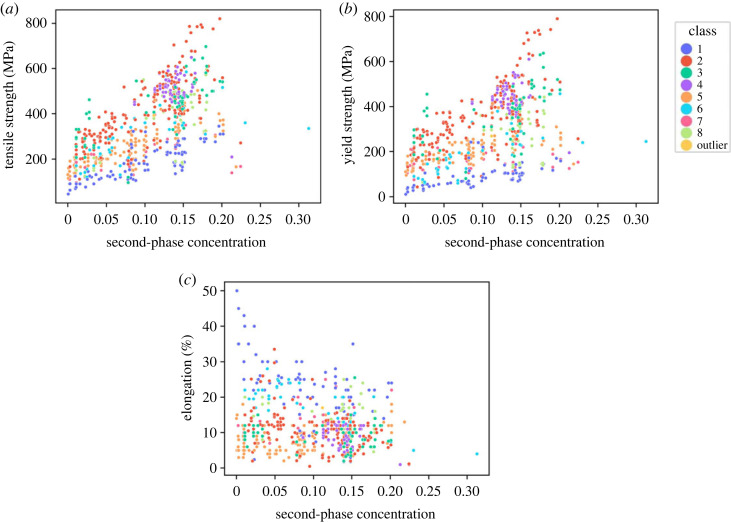
Mechanical property variation with the second-phase concentration. (*a*) Tensile strength versus wt fraction second phase, (*b*) yield strength versus wt fraction second phase, (*c*) elongation versus wt fraction second phase.

Figure 12 shows the variation of the three mechanical properties with the concentration of the second phase, calculated using PANDAT® [37]. PANDAT is an implementation of the CALPHAD approach that uses equilibrium thermodynamics to predict alloy phase fractions (for a given composition). The properties were calculated at 25° Celsius at equilibrium conditions. Such an approach permits (without the need to source alloys and carry out experiments) an estimation of the proportion of the second phase that may be found in any given alloy. Once known, that alloy characteristic can also be explored in selecting the class of desired alloys, while designing Al alloys when there are restrictions on second-phase concentration due to design requirements.

The sorting of Al alloys into classes is promising from a design perspective since, depending on the end requirements, attention could be focused on the relevant class that encompasses the currently used or incumbent alloys. For example, the structural alloys for aircraft notionally belonged in Class 4, while automotive and marine alloys (i.e. 6xxx, 5xxx series alloys) were typically observed in Class 2.

ML models, trained to predict the mechanical properties of the alloys, have assisted in alloy design [13,52,53]. The accuracy of such ML models can be improved by separating the dataset into classes and training individual models for each class [54,55]. Traditionally, this class separation has been based on properties with the help of domain knowledge [55]. This study provides an alternative where the classes are detected based on feature similarity. UL classes based on feature similarity have been shown to be more separable than SL property-based classes in silicon quantum dots [56]. Further, the regression model trained on UL classes to predict emission wavelength was more accurate than the model trained on supervised property labels. Hence, individual models trained on individual Al-alloy classes can potentially have higher accuracy compared with a model trained on the entire dataset.

This level of interpretability not only provides illumination on existing alloy classes but also is the requisite precursor for the development of ML models for individual classes that may be used for predicting new alloys. Computationally and from the perspective of mechanistic interpretability, searching for new alloys within one class is a more sustainable and achievable goal than searching through the entire feature space, as it facilitates the choice and planning of resources, processing methods and equipment. The classes can further help the development of specific ML models for a smaller subset of alloys, rather than designing a model of the entire feature space of Al alloys. This could allow for more customization of models and higher accuracy.

## 5. Conclusion

The objective of the present study was to show that an unsupervised clustering algorithm can be used to detect separable classes based only on feature similarity. An unsupervised ML approach was successfully applied to identify the underlying (hidden) patterns and to label alloys accordingly into classes. The prediction of the different classes of Al alloys, drawing from a dataset of 1154 Al alloys, included alloy composition and processing conditions. Using the ILS approach, eight unique clusters of Al alloys were identified based on the feature set of 34 dimensions. Using a DTC, these clusters were shown to be separable classes. It was determined that only 11 features played an essential role in determining the classes using recursive feature elimination using a DTC. The feature importance profiles found that processing conditions and major alloying combinations primarily separated the eight classes. It was shown that the approach was capable of extracting information regarding Al-alloy classes that is interpretable and which meaningfully discriminated some of the major classes of commercial alloys in a rational manner. Based on the work herein, the classes may serve as a guide for rational, data-driven Al-alloy design in future models that seek to optimize alloy design using ML.

## Data Availability

The raw data required to reproduce the results are available to download from [32]. The information about mechanical properties of the alloys can be requested from the corresponding author.
